# Abiotic polycyclic aromatic hydrocarbons originating from the sub-oceanic mantle

**DOI:** 10.1038/s41598-025-32798-x

**Published:** 2026-01-14

**Authors:** Itaru Mitsukawa, Akira Miyake, Yohei Igami, Tetsu Kogiso, Norikatsu Akizawa, Junya Matsuno, Megumi Matsumoto, Akira Tsuchiyama, Kentaro Uesugi, Masahiro Yasutake, Tomoki Taguchi, Yoshio Takahashi, Shohei Yamashita, Shota Okumura

**Affiliations:** 1 Division of Earth and Planetary Sciences, Graduate School of Science, Kyoto University, Kitashirakawaoiwake-cho, Sakyo-ku, Kyoto-shi, Kyoto, 606-8502 Japan; 2 Graduate School of Human and Environmental Studies, Kyoto University, Yoshida-nihonmatsu-cho, Sakyo-ku, Kyoto, 606-8501 Japan; 3 Graduate School of Advanced Science and Engineering, Hiroshima University, 1-3-2 Kagamiyama, Higashihiroshima-shi, Hiroshima, 739-8526 Japan; 4 Department of Earth and Planetary Materials Sciences, Tohoku University, 6-3, Aramaki-Aza-Aoba, Aoba-ku, Sendai-shi, Miyagi, 980-8578 Japan; 5 Research Organization of Science and Technology, Ritsumeikan University, 1-1-1 Noji-higashi, Kusatsu-shi, Shiga, 525-8577 Japan; 6 Key Laboratory of Mineralogy and Metallogeny, Guangdong Provincial Key Laboratory of Mineral Physics and Materials, Chinese Academy of Sciences (CAS), Guangzhou Institute of Geochemistry, CAS, Guangzhou, 510640 China; 7 CAS Center for Excellence in Deep Earth Science, Guangzhou, 510640 China; 8 Spectroscopy and Imaging Division, Japan Synchrotron Radiation Research Institute (JASRI/SPring-8), 1-1-1, Kouto, Sayo-cho, Sayo-gun, Hyogo, 679-5198 Japan; 9 Department of Earth Sciences, Faculty of Education and Integrated Arts and Sciences, Waseda University, 1-6-1, Nishi-waseda, Shinjuku-ku, Tokyo, 169-8050 Japan; 10 Department of Earth and Planetary Science, The University of Tokyo, 7-3-1, Hongo, Bunkyo-ku, Tokyo, 113-0033 Japan; 11 Institute of Materials Structure Science Photon Factory, High Energy Accelerator Research Organization (KEK), 1-1 Oho, Tsukuba-shi, 305-0801 Ibaraki Japan

**Keywords:** Biogeochemistry, Planetary science, Solid Earth sciences

## Abstract

Experimental studies have demonstrated that petroleum may form from abiotic organic compounds synthesised in the Earth’s upper mantle. However, the most direct evidence of mantle-derived organics, as obtained from whole-rock analyses, indicates ambiguous origins. Although inclusions containing organic matter have been reported in minerals from kimberlite pipes, such occurrences have not previously been documented in mantle xenoliths derived from the sub-oceanic upper mantle, which represent the predominant lithology of the Earth’s upper mantle. In this study, we identified polycyclic aromatic hydrocarbons (PAHs)-bearing melt inclusions within a spinel-bearing unaltered harzburgite xenolith from Tahiti Island. The inclusions consisted of platinum-group minerals, base metal sulphides, silicate glass, and C–O–H phases. Our comprehensive sub-micrometre-scale analyses revealed the presence of PAHs in the C–O–H phases. These PAHs contained few functional groups or aliphatic chains and coexisted with CO and CO₂. They likely formed under reducing conditions in deeper regions of the upper mantle without serpentinisation. Our findings demonstrate abiotic organic matter formation in the sub-oceanic mantle and suggest widespread abiotic organic synthesis in the sub-oceanic mantle.

**Supplementary Information:**

The online version contains supplementary material available at 10.1038/s41598-025-32798-x.

## Introduction

Petroleum is most commonly explained by the biogenic theory, which posits that it originates from biological material. Kerogen, a complex, insoluble substance primarily formed from the remains of algae and terrestrial plants, is transformed into petroleum over millions of years under heat and pressure^1^. In contrast, the abiogenic theory proposes that hydrocarbon fluids are generated in the Earth’s upper mantle without complex organic precursors or biological activity^2^. According to this theory, such hydrocarbons, referred to as “abiotic” or “abiogenic,” migrate upward along deep-seated faults and accumulate in shallow reservoirs^2^. Experimental studies have demonstrated natural gas and liquid hydrocarbon synthesis from inorganic compounds in the upper mantle under high-temperature and high-pressure conditions^3,4^. Moreover, under such conditions, hydrocarbons tend to polymerise into heavier species rather than undergo thermal cracking^5–8^. Collectively, these findings suggest that complex organic molecules could plausibly form in the Earth’s upper mantle.

The most direct evidence for abiogenic organic synthesis in the mantle would be organic matter in mantle-derived materials. Sugisaki and Mimura^9^ performed gas chromatography–mass spectrometry (GC–MS) on various igneous rocks and detected saturated hydrocarbons ranging between C_14_ and C_33_, exclusively in mantle xenoliths and tectonites. Additionally, diverse organic compounds, such as aliphatic, cyclic, and oxygenated hydrocarbons, have been reported in minerals from kimberlite pipes based on GC–MS analyses^10,11^. Although these results suggest the presence of organic matter in mantle rocks, whole-rock analyses do not reveal the specific microstructural contexts of these compounds. Therefore, the origin of such organic matter remains unclear. Although the detection of organic matter within fluid or melt inclusions is critical in confirming their mantle origin, such inclusions have rarely been reported in kimberlite pipes^12,13^ and ultrahigh-pressure (UHP) peridotites^14^. Notably, such evidence has not been documented in mantle xenoliths from the sub-oceanic upper mantle, which constitutes the majority of the Earth’s upper mantle. This lack of evidence supports the prevailing view that conditions for deep hydrocarbon generation are not uniformly available throughout the mantle, potentially explaining the limited distribution of petroleum resources^2^.

In this study, we identified melt inclusions containing polycyclic aromatic hydrocarbons (PAHs) within a clinopyroxene (Cpx) grain from a spinel-bearing harzburgite xenolith (sample H3–001_TK) collected from Tahiti Island, part of the Society hotspot track^15^. We conducted detailed analyses of the submicrometre melt inclusions using X-ray nano-computed tomography (XnCT), Raman microscopy, wide-field fluorescence microscopy, and X-ray absorption near-edge structure (XANES) spectroscopy with scanning transmission X-ray microscopy (STXM) (Supplementary Fig. S1). This study provides the first clear evidence of abiotic PAHs formation in fresh peridotite xenoliths from the sub-oceanic mantle.

## Results

**Fig. 1:**
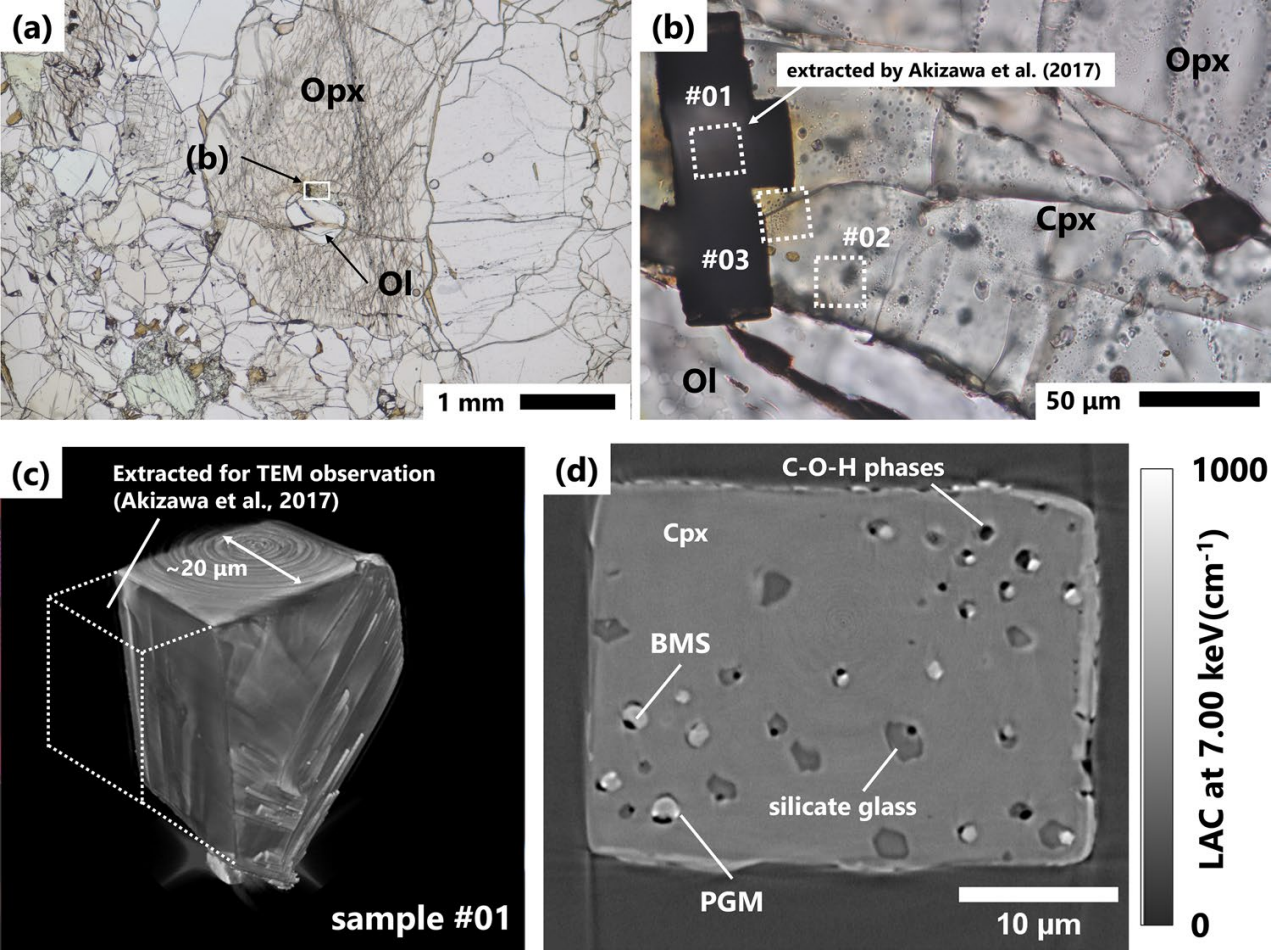
(a) Photomicrograph of the host Cpx grain in thin section “H3–001_TK.” The white rectangle indicates the area shown in (b). (b) Magnified image of the host Cpx grain. The white-dotted rectangles indicate regions from which samples for detailed analyses were extracted using a focused ion beam (FIB). The numbers adjacent to the rectangles indicate the sample numbers. PGMs-containing inclusions were observed only in the Cpx grain. Three samples were extracted to ensure that these inclusions were included. (c) 3D view of sample #01, shown as a representative block sample analysed in this study. The image was reconstructed from X-ray nano-computed tomography data. White-dotted lines indicate the portion of sample #01 previously extracted for transmission electron microscopy (TEM) analyses [15]. (d) Cross-sectional image obtained by X-ray nano-computed tomography. Abbreviations: Ol = olivine; Opx = orthopyroxene; Cpx = clinopyroxene; PGM = platinum-group minerals; BMS = base metal sulphides; STXM–XANES = X-ray absorption near-edge spectroscopy combined with scanning transmission X-ray microscopy.

The Cpx grain was attached to an olivine grain and enclosed by a large orthopyroxene (Opx) grain (approximately 3 mm in diameter) (Fig. 1a–b). Cpx is a Na-rich diopsidic augite that exhibits slightly different elemental compositions compared to other Na-rich Cpx grains in the same thin section (Supplementary Fig. S2 and Supplementary Table S1). Platinum-group minerals (PGMs) have been identified within the Cpx grain using transmission electron microscopy (TEM)^15,16^ while the surrounding grains do not contain PGMs. This anomalous feature motivated a detailed investigation of the inclusions in the Cpx grain. One house-shaped and two cylindrical samples (#01, #02, and #03) were extracted from the Cpx grain using a focused ion beam system combined with scanning electron microscopy (FIB–SEM) (Fig. 1b–c; Supplementary Figs. S3 a–b). The samples were subsequently analysed using XnCT to reconstruct the 3D arrangement of the constituent phases in the inclusions (Fig. 1d; Supplementary Figs. S4–6). After further sample preparation using FIB-SEM (Supplementary Fig. S3), the inclusions were analysed using Raman microscopy, wide-field fluorescence microscopy, and STXM–XANES (Supplementary Fig. S1). Notably, 500 nm-thick foils along the inclusion arrays were extracted from samples #01 and #02 using FIB-SEM (Supplementary Fig. S3a–b).

In each sample, four phases were identified as inclusions in the XnCT images (Fig. 1d; Supplementary Figs. S4–6). Based on TEM analyses^15,16^, the phases were identified as PGMs (Ir–Pt–Rh–Cu sulphide), base metal sulphide (BMS) (Fe–Ni–Cu sulphides), silicate glass, and C–O–H phases. The inclusions can be classified into the following groups: (a) C–O–H, (b) silicate glass + C–O–H, (c) PGMs + BMS + C–O–H, and (d) PGMs + BMS + silicate glass + C–O–H. Notably, all inclusions contained C–O–H phases.

**Fig. 2:**
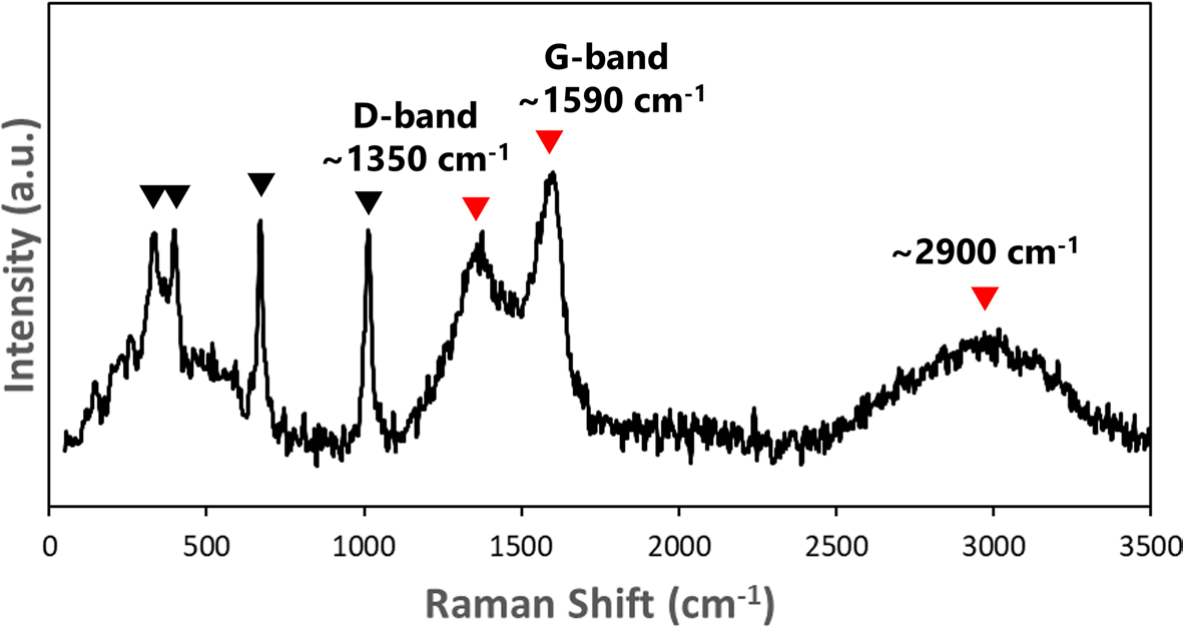
Raman spectrum obtained from an inclusion in sample #01. Red and black triangles indicate peaks derived from C–O–H phases and host Cpx, respectively. Similar spectra were obtained from seven inclusions in sample #01 and one inclusion in sample #02. Abbreviation: a.u. = arbitrary units.

**Fig. 3:**
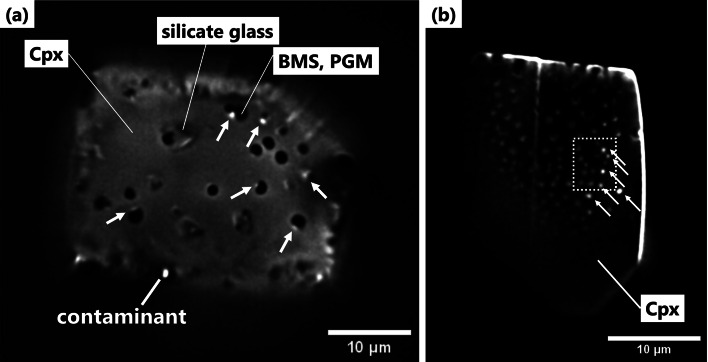
Wide-field fluorescence images obtained using the I3 filter (excitation wavelength: 450–490 nm; detection wavelength: > 515 nm). White arrows indicate C–O–H phases exhibiting strong fluorescence. (**a**) Sample #01; (**b**) Sample #02. The white-dotted rectangle in (b) marks the area shown in Fig. 4. Fluorescence was observed with three dichroic mirrors (D, I3, and N21) in Supplementary Table S2. Abbreviations: Cpx = clinopyroxene; BMS = base metal sulphides; PGM = platinum-group minerals.

Raman spectra from the C–O–H phases in samples #01 and #02 exhibited two broad peaks at approximately 1,590 cm⁻¹ (G-band) and 1,350 cm⁻¹ (D-band), along with a broad peak at approximately 2,900 cm⁻¹ (Fig. 2; Supplementary Fig. S7). These spectral features resemble those of amorphous carbonaceous matter, primarily composed of PAHs^17^, such as bitumen^18^, coal^19^, kerogen^20^, and diesel soot, or industrial carbon black^21^. Furthermore, in the wide-field fluorescence images of samples #01 and #02, fluorescent light was detected in the C–O–H phases of the inclusions using three dichroic mirrors: D, I3, and N21 (Fig. 3 and Supplementary Table S2). These fluorescence features were similar to those of bitumen samples^22^.

**Fig. 4:**
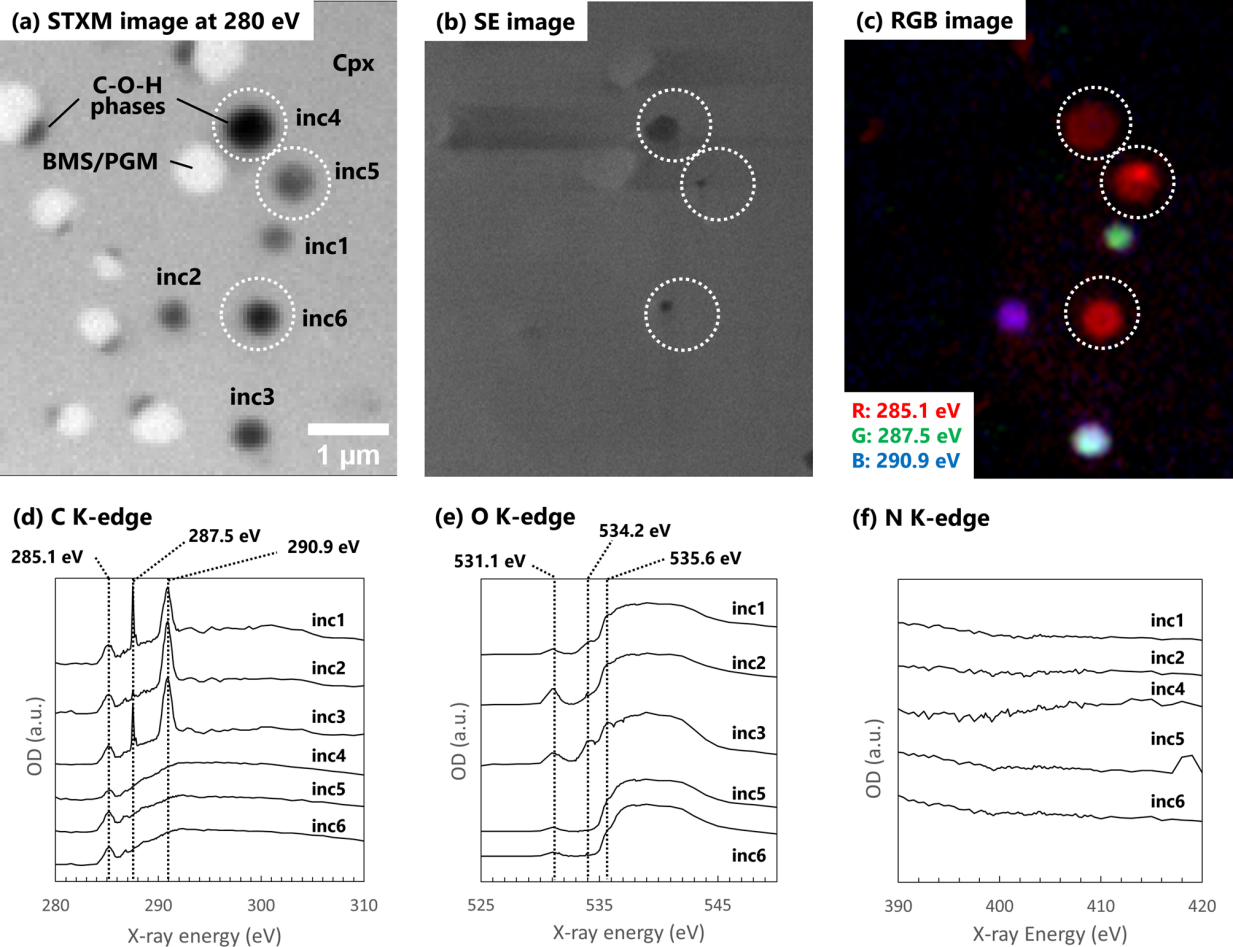
Results of STXM–XANES analyses for sample #02. (**a**) STXM image at 280 eV, where contrast corresponds to the degree of absorption. The numbers beside each inclusion correspond to those in (**d**), (**e**), and (f). White-dotted circles indicate the inclusions that did not exhibit peaks at 287.5 and 290.9 eV and correspond to those in (**b**) and (**c**). (**b**) Secondary electron (SE) image of the sample surface. Notably, inclusions indicated by white-dotted circles are exposed on the sample surface. (**c**) RGB image. Each colour corresponds to the integrated absorption signals of the peaks: red = 285.1 eV, green = 287.5 eV, and blue = 290.9 eV, in the C K-edge XANES spectra. Notably, the colour variations in non-exposed inclusions are primarily attributed to differences in their CO/CO_2_ ratios. (d) C K-edge XANES spectra. (e) O K-edge XANES spectra. Peaks at 531.1 eV are attributed to oxygen in silicate glass. (**f**) N K-edge XANES spectra. Spectra shown in (d–f) were obtained by subtracting the normalised host Cpx spectrum from those of the inclusions. Abbreviations: OD = optical density; a.u. = arbitrary units; Cpx = clinopyroxene; BMS/PGM = base metal sulphides and platinum-group minerals.

Furthermore, the STXM–XANES analyses detected PAHs, CO, and CO_2_ in the C–O–H phases within the inclusions (Fig. 4; Supplementary Fig. S8). A peak at 285.1 eV was consistently observed in the C-XANES spectra extracted from the C–O–H phases (Fig. 4d and Supplementary Fig. S8d). This peak is attributed to aromatic carbon or carbon atoms that form C = C bonds^23^. Furthermore, the STXM-XANES analyses revealed that the PAHs in the inclusions contained few functional groups or aliphatic chains (Fig. 4d–f; Supplementary Fig. S8d–f). The XANES spectra of the inclusions did not show any significant peaks corresponding to aliphatic chains or functional groups. Some C–O–H phases of the inclusions not exposed to the outside exhibited peaks at 287.5 and 290.9 eV in the C–XANES spectra and those at 534.2 and 535.6 eV in the O–XANES spectra (Fig. 4d–e; Supplementary Fig. S8d–e). These species are interpreted as components of fluid phases. The peaks at 287.5 and 534.2 eV can be attributed to CO while those at 290.9 and 535.6 eV can be attributed to CO_2_^24,25^. No significant edges or peaks were observed in the N-XANES spectra (Fig. 4f; Supplementary Fig. S8f), indicating that the C–O–H phase did not contain nitrogen.

## Discussion

### Inclusion formation processes

The PAHs detected in this study were not biological contaminants because their signatures were extracted from inclusions fully enclosed within the Cpx grain. PAHs signals were consistently detected in all samples using Raman microscopy, fluorescence imaging, and STXM-XANES analyses. Surface contamination may occur either during or after sample preparation. However, it is distinguishable whether the signal originates from the inclusions or from surface contaminants by verifying whether the signal is obtained exclusively from within the inclusions or also from inclusion-free areas. Raman microscopy and STXM–XANES analyses were performed multiple times for each inclusion; however, the spectral features remained consistent. Thus, PAHs, CO, and CO_2_ likely formed independently of biotic activity or artificial alterations during the analytical procedures.

**Fig. 5:**
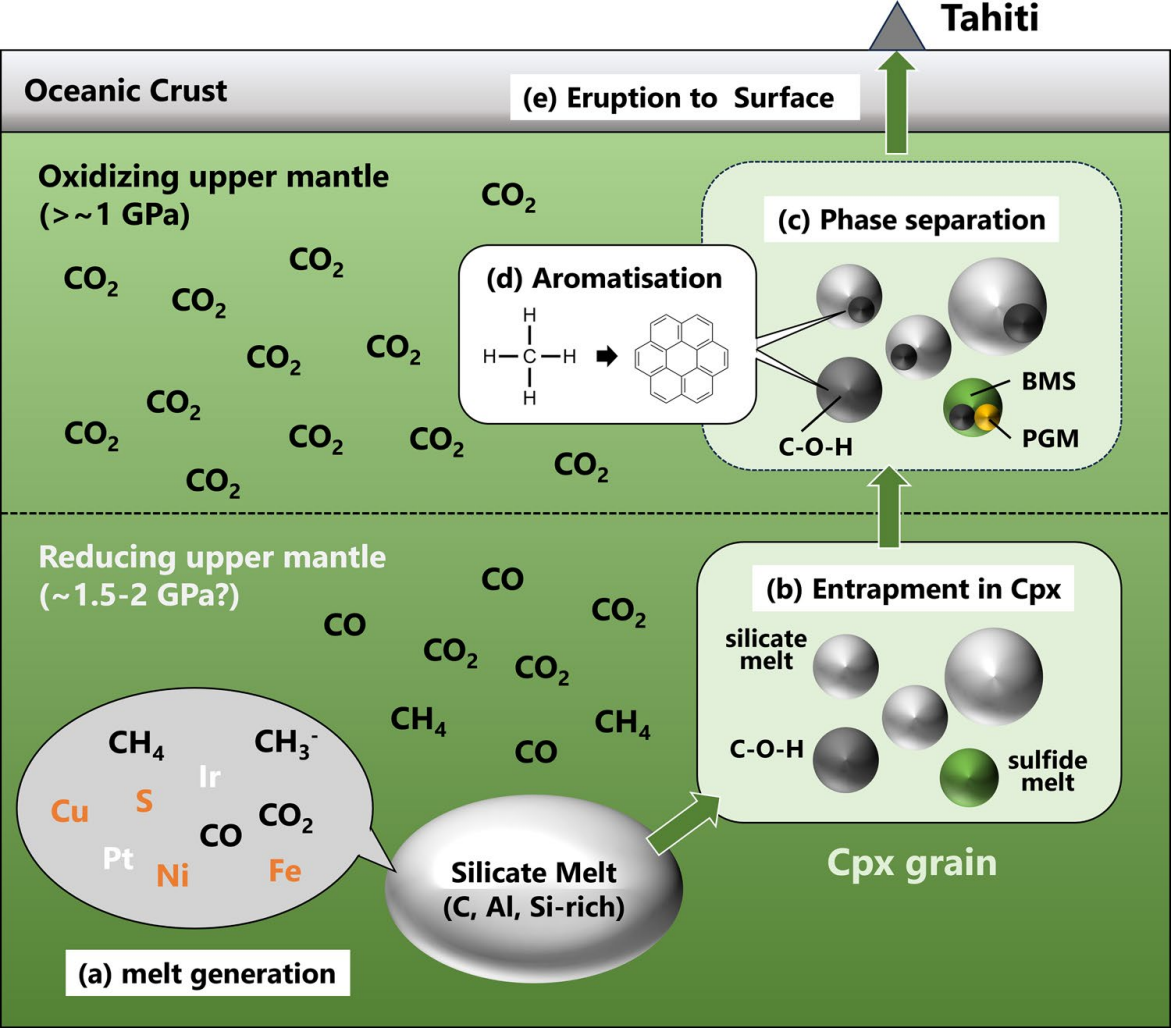
Schematic illustration of the proposed inclusion formation model. (**a**) Generated melt was enriched in C, Al, and Si, containing dissolved C–O–H species, such as CH_4_, CO, and CO_2_, along with Fe, Ni, Cu, PGEs (Ir, Pt, and Rh), and S, based on the results of Akizawa et al. ^15^. (**b**) The melt is trapped by the Cpx grain, separated into three immiscible phases: silicate melt, sulphide melt, and C–O–H phases. (**c**) After the melt was trapped in individual inclusions, the C–O–H phase was expelled due to decompression. PGMs and BMS crystallized at temperatures above 1000 ℃. The inclusions remained protected from oxidation in the shallower mantle. (**d**) Aromatisation of simple C–H molecules occurred in C–O–H fluids. (**e**) The peridotite was subsequently transported to the surface. Abbreviation: Cpx = clinopyroxene.

The inclusions investigated herein were aligned along a plane within the Cpx grain (Fig. 1; Supplementary Figs. S4–6), and this plane may have formed either through microcracking during secondary melt infiltration or by decrepitation of the initially trapped melt. The cracks later healed, trapping the inclusions. In any case, the melt-trap in the Cpx occurred under high-temperature and high-pressure conditions in the upper mantle, as the crystallization temperature of sulphide minerals is estimated to be above 1000°C^16^. According to high P–T experiments (< 1,700 ℃ and < 4 GPa), carbon speciation in melts strongly depends on redox conditions^26–30^. Under oxidising conditions, carbon exists primarily as CO_2_ or CO_3_^2–^ in silicate melts^26^. In contrast, under reducing conditions, CO, CH_4_, CH_3_^–^, and molecules containing –C≡C–H, atomic C, or amorphous C have been identified in quenched silicate glasses^27–30^. Therefore, reducing conditions are required for the formation of PAHs and CO; these reduced species are likely precursors of PAHs (Fig. 5a).

At oxygen fugacities more oxidizing than one log unit below the fayalite–magnetite–quartz buffer (FMQ−1), carbon is thought to occur predominantly as CO_2_^31^. Hence, the presence of reduced carbon species such as CO or PAHs is considered to require fluid entrapment under more reducing conditions. The redox states of the sub-continental mantle are known to decrease with depth^32^. In spinel-bearing abyssal peridotites, which represent the oceanic mantle lithosphere, measured oxygen fugacity values ranging from FMQ to FMQ2.5^32^. Therefore, considering that the host-rock is spinel-bearing peridotites, we propose that this inclusion was trapped in the deeper part of the spinel stability field (~ 1.5 to 2 GPa) (Fig. 5b). Variable Fe^3+^/Fe^2+^ values of spinel (0.09 to 0.41) have been reported for mantle xenoliths from Tahiti Island^15^, indicating that the rocks have experienced variable redox conditions. This chemical characteristic supports the interpretation that the Cpx grain was once emplaced in a reducing environment at greater depth.

Previous experimental studies have indicated that carbon solubility in melts increases with pressure^33,34^. Thus, the C–O–H molecules that subsequently formed the C–O–H phases were likely separated from the melts in response to the pressure decrease during the formation of the inclusions (Fig. 5b–c). PAHs were detected only in the C–O–H phases in the inclusions; thus, PAHs likely formed after separation of the C–O–H phases at pressures below approximately 2 GPa (Fig. 5b–c). Although experimental studies on PAHs formation under geological conditions are limited and the mechanisms remain poorly understood, we propose that PAHs formed through aromatisation reactions under upper mantle conditions, as several studies have suggested the feasibility of such reactions^8,35,36^ (Fig. 5d). For example, high-P–T experiments at 850 K and 2.5 GPa demonstrated benzene ring formation from methane via cyclisation reactions^8^. Notably, longer experimental durations (approximately 10 h) promoted the formation of more complex hydrocarbons^8^. This suggests the possibility of larger molecule formation, including PAHs, through these reactions on a geological timescale. Likewise, high P–T pyrolysis experiments (300–700 ℃, 1–3 GPa) reported that pressure favours the aromatisation of pyrolysates^36^. Thermodynamic calculations showed that aromatic compounds dominated C–O–H fluids at 5–8 GPa and 1,400–2,000 K^35,37^. Additionally, the catalytic effects of the host minerals (Cpx) or coexisting phases (BMS, PGMs, silicate glasses, CO, and CO_2_) may facilitate these reactions. Olivine and pyroxene act as catalysts for PAHs formation^38,39^. PGMs and BMS may also act as catalysts for PAHs formation^40^. However, PAHs are also observed in inclusions that lack these minerals (Fig. 4), suggesting that their catalytic effects are not necessarily required for PAHs formation. Further experimental and theoretical studies on PAHs formation conditions, focusing on reaction kinetics and mineral catalysis, would be crucial for revealing the universality of PAHs formation in the upper mantle.

### Implications of abiotic petroleum formation in the Earth’s upper mantle

Abiotic organic matter within the oceanic lithosphere has primarily been reported in rocks from subduction zones and mid-ocean ridges. In these settings, H_2_-rich fluids generated by serpentinisation create strongly reducing conditions that promote the abiotic synthesis of diverse organic compounds, including methane, short-chain hydrocarbons, organic acids^31^, and carbonaceous matter, which are predominantly composed of PAHs^41,42^. Serpentinisation has therefore been widely regarded as a prerequisite for abiotic organic synthesis in the sub-oceanic lithosphere; such synthesis has been considered largely confined to subduction zones and mid-ocean ridges. However, our findings demonstrate abiotic PAHs formation occurring independent of serpentinisation. This conclusion is supported by these observations: (1) the analysed peridotite exhibited no petrographic or mineralogical evidence of serpentinisation^15^; (2) sulphide crystallisation temperatures within the same inclusion array were estimated at approximately 1,000 ℃^16^—well above the thermal range of serpentinisation; and (3) the fluid phase associated with the inclusions contained CO and CO_2_, rather than the CH_4_ and H_2_ typically found in serpentinised systems, indicating a distinct formation environment. These results indicate that strongly reducing conditions conducive to abiotic organic synthesis exist in deeper portions of the sub-oceanic upper mantle without serpentinisation. The identified PAHs provide robust evidence for abiotic organic synthesis in the sub-oceanic upper mantle and expand their spatial domain to encompass the entire region.

Experimental and theoretical studies have shown temperature and pressure effects on the characteristics of organic compounds formed in the mantle^37^. Hydrocarbons tend to evolve from saturated to unsaturated or aromatic forms with an increase in temperature^37^. The mantle beneath Tahiti Island may exhibit a higher geothermal gradient as the mantle plume has been inferred by seismic imaging techniques^43^. Accordingly, the predominance of aromatic structures may reflect the elevated temperatures of the mantle beneath Tahiti Island. Due to limited presence of aliphatic moieties, these PAHs have a low potential for generating natural gas or petroleum. In contrast, the majority of the sub-oceanic mantle is characterised by lower temperatures, under which the formation of aliphatic-rich organic matter is anticipated. Therefore, the formation of aliphatic-rich mantle-derived “kerogen” or petroleum itself in the sub-oceanic upper mantle is a plausible scenario. The saturated hydrocarbons detected by GC–MS analysis in Sugisaki and Mimura^9^ may represent mantle-derived aliphatic-rich hydrocarbons if located within inclusions and are mantle-derived.

Fluid inclusions in fresh peridotite xenoliths, including those from Tahiti, are generally thought to be dominated by pure CO_2_^15,44–46^, which reflects the oxidising conditions of the uppermost mantle. In contrast, our findings demonstrated that abiotic PAHs can be retained within melt inclusions in peridotite xenoliths. Although such organic matter-bearing inclusions are rare, they can serve as key tracers for assessing the possibility of abiogenic petroleum formation throughout the mantle. If abiotic organic synthesis is widespread in the sub-oceanic upper mantle, current estimates of the Earth’s petroleum reserves and the abundance of organic matter may require substantial revision. Future research should aim to uncover a hidden inventory of deep-Earth organic matter to redefine our understanding of the planet’s carbon cycle and the origin of petroleum.

## Methods

### FE-SEM-EDS analysis and sample preparation using FIB–SEM

The elemental compositions of the Cpx grains in the thin section were determined using a scanning electron microscope equipped with a field-emission electron gun (FE-SEM, JEOL JSM-7001 F, Tokyo, Japan) and an energy-dispersive X-ray spectrometer (EDS, Oxford Instruments, X-MAX^150^, England, UK) at Kyoto University. X-ray spectra were collected for 180 s at each point. The analytical conditions were set at an accelerating voltage of 15 kV and a probe current of 0.35 nA. The obtained X-ray spectra were analysed to quantify the elemental compositions using the ‘AZtec’ analytical software (Oxford Instruments).

One house-shaped sample (#01) and two cylindrical samples (#02 and #03) (20–30 μm) were extracted from the Cpx grain using FIB–SEM (Helios NanoLab G3 CX or Quanta 200 3DS, Thermo Fisher Scientific, Waltham, MA, USA) at Kyoto University (Fig. 1 and Supplementary Fig. S3a–b). All three samples contained nano-inclusion arrays. Each sample was mounted on a W-needle (sample #01) or a Ti-needle (samples #02 and #03). To capture the three-dimensional geometry of the internal inclusions, XnCT experiments were performed at BL47XU of SPring-8, a synchrotron facility in Hyogo, Japan^47^.

Based on the XnCT images, each sample was further trimmed using an FIB–SEM system. For Raman and wide-field fluorescence microscopy, sample #01 was removed from the W-needle and placed on a carbon-coated glass slide, such that the inclusion array was oriented horizontally (Supplementary Fig. S3c). The upper surface of the sample was polished using Ga^+^ ion beams at an accelerating voltage of 30 kV and beam current of 0.40–2.5 nA. Additionally, two microslab samples were extracted from Samples #02 and #03 for Raman, fluorescence, and STXM–XANES analyses (Supplementary Fig. S2a, b, and d). Samples #02 and #03 were removed from the Ti-needle, attached to Ti grids by Pt deposition, and thinned into 500 nm-thick foils along the plane on which the targeted inclusions were aligned. Thinning was performed at an accelerating voltage of 30 kV and a beam current of 0.77–9.4 nA. After thinning, the surfaces of the samples were treated with Ga^+^ ion beams at 2 kV and 0.77 nA to remove damaged layers. Although a thickness of approximately 500 nm is generally considered too large for STXM–XANES analyses, it was found acceptable for identifying the X-ray absorption in the inclusions. More importantly, nearly the entire targeted inclusion array was enclosed within a slab of this thickness. The sample numbers for the three microsamples and the analyses performed are summarised in Supplementary Fig. S1. The detailed analytical conditions are described below.

### Synchrotron X–ray nano computed tomography (XnCT)

The three-dimensional distribution of melt inclusions in Cpx was investigated using XnCT experiments at beamline BL47XU of SPring-8, a synchrotron facility in Hyogo, Japan. Dual-energy tomography (DET) was performed at X-ray energies of 7.00 and 7.35 keV^48^. This technique enables visualisation of the spatial distribution of the linear absorption coefficient (LAC) within the sample at two X-ray energies. The sample was rotated in 0.1° increments per projection; 1,800 projections were acquired with an exposure time of 500 ms each. Cross-sectional images were reconstructed using a convolution back projection algorithm. The voxel size of the reconstructed images was in the range of 15–50 nm. CT data were analysed using the ‘*Slice*’^49^ and ‘*ImageJ*’^50^ analytical software.

### Investigations of C–O–H phases in the inclusions

Raman microscopy was performed on the inclusions of samples #01 and #02. The spectra were obtained using a confocal laser Raman microscope (DXR3, Thermo Fisher Scientific, Waltham, MA, USA) at Waseda University. Each spectrum was collected for 60–200 s using a 532 nm green laser at a power of 0.3–6 mW. The laser was focused through a ×50 objective lens. Raman scattered light was collected through a 25 μm pinhole, gratings of 400, 900, and 1,800 lines/mm, and detected by a charge-coupled device camera. The background of the spectra was subtracted using analytical software (Supplementary Fig. S6).

Wide-field fluorescence microscopy of samples #01 and #02 was performed using a fluorescence microscope (Leica Microsystems, THUNDER Imaging System, Wetzlar, Germany) in the Leica Microsystems demonstration room in Tokyo. The wavelengths of the excitation and detected light were controlled using dichroic mirrors. Sample #01 was examined using five filters; sample #02 was analysed using an I3 filter (Supplementary Table S1). Background signals from out-of-focus regions were removed using a computational clearing method to improve the signal-to-noise ratio (S/N) of the images^51^.

STXM–XANES was conducted for carbon, oxygen, and nitrogen K-edges XANES (C, O, and N K-edge XANES) for samples #02 and #03 at beamline BL-19 A, Photon Factory, High-Energy Accelerator Research Organisation (KEK) in Tsukuba, Japan^52^. Synchrotron X-rays were monochromatized using a grating and focused using Fresnel zone plate (FZP) optics. An order-sorting aperture (OSA) was used to eliminate high-order diffraction light, set to 40–50 μm in width and height. The spot size of the soft X-ray beam at the focal point (i.e., on the sample) was approximately 35 nm. The sample was then moved to a 2D plane with a step size of 50 nm. C, O, and N K-edge XANES spectra were obtained in the energy ranges of 280–310, 525–550, and 390–415 eV, respectively. The energy step size was 0.1–1.0 eV. The acquisition time per image pixel for each energy step was 10–40 ms. The intensity of the incident X-ray beam (I_0_) was measured during or after obtaining the spectral image stacks of the samples. The spectral signal (I) was converted to ln(I/I_0_) (optical density, OD) after drift correction using Sobel and double-Hanning window filters. Subsequently, the inclusion spectra were retrieved by subtracting the normalised Cpx spectra.

## Supplementary Information

Below is the link to the electronic supplementary material.

Supplementary Material 1

## Data Availability

The datasets generated during and/or analysed during the current study are available from the corresponding author on reasonable request.
