# Supplementary material for: Abiotic polycyclic aromatic hydrocarbons originating from the sub-oceanic mantle
Source: Sci Rep. 2026 Jan 14;16:638. doi: 10.1038/s41598-025-32798-x (PMC12804844; doi:10.1038/s41598-025-32798-x)
Supplement: Supplementary file 1 — Supplementary Material 1 [file 41598_2025_32798_MOESM1_ESM.docx]

**Supplementary Figures**


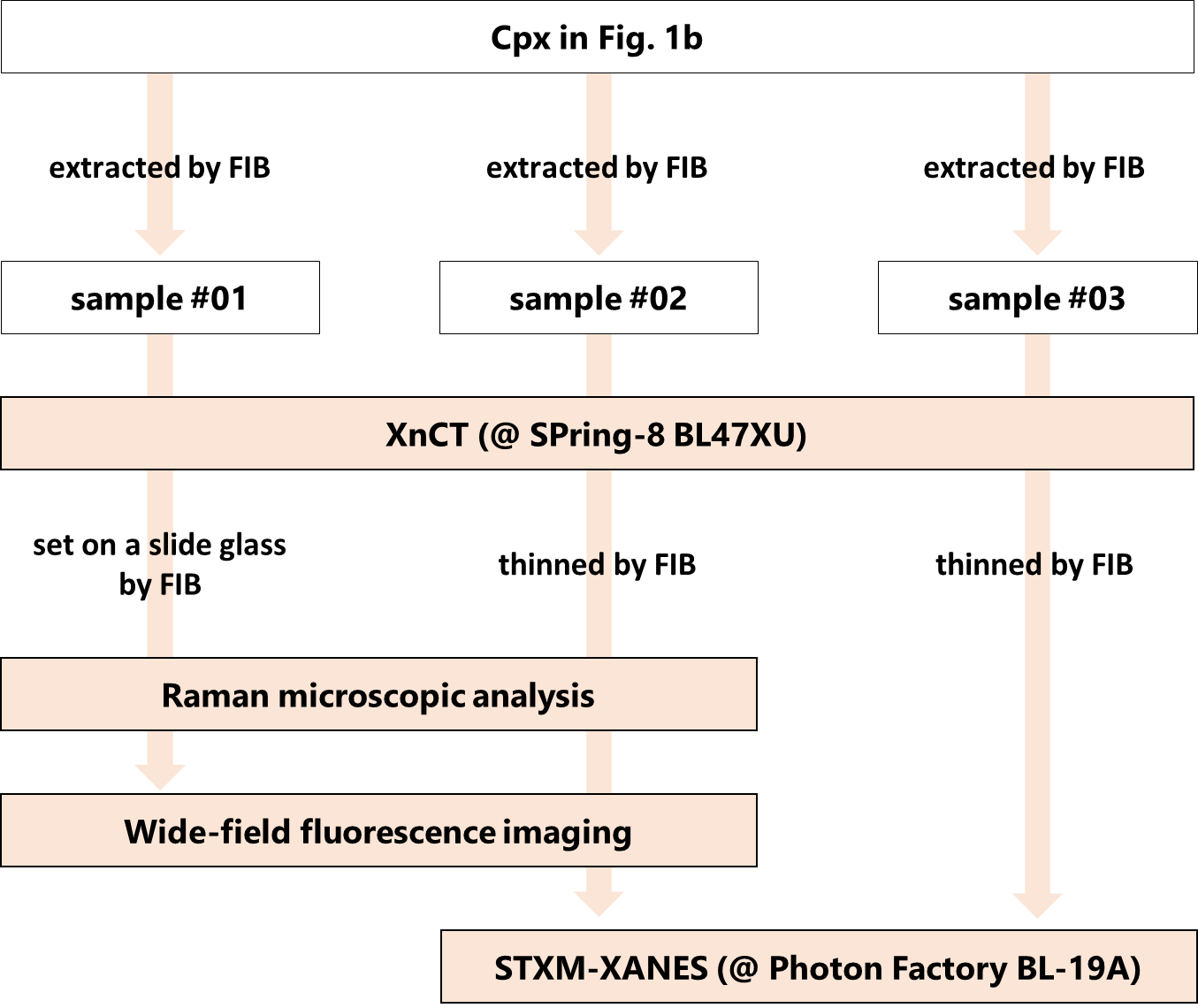


Supplementary Fig. S1. Summary of the study analyses. Abbreviations: STXM–XANES, X-ray absorption near-edge spectroscopy (XANES) with scanning transmission X-ray microscopy (STXM); FIB, focused ion beam; XnCT, X-ray nano-computed tomography.


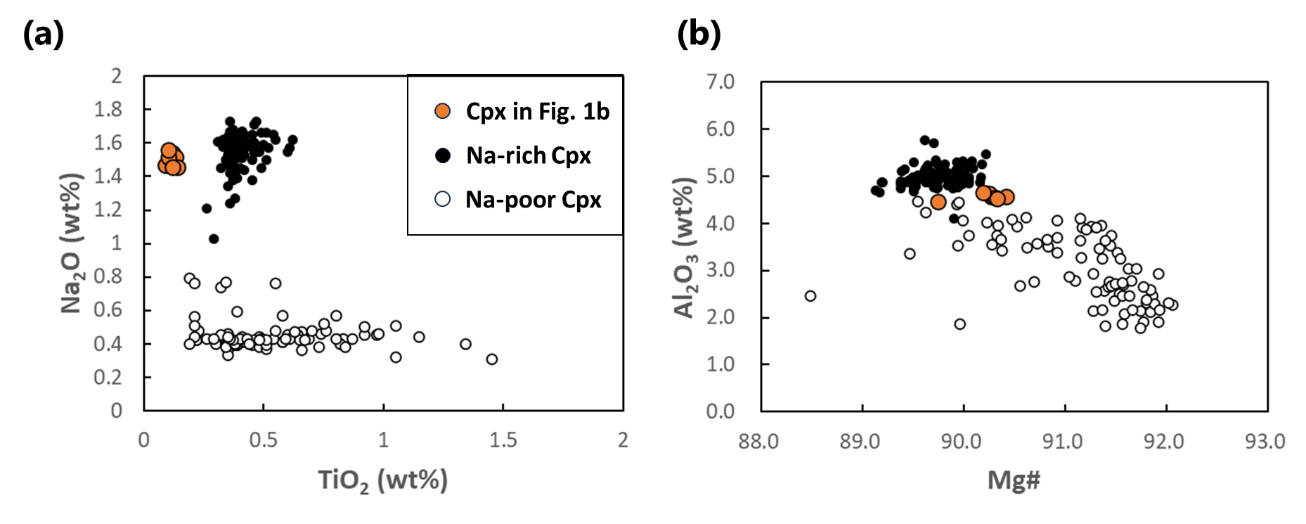


Supplementary Fig. S2 Elemental compositions of Cpx grains in the thin section “H3-001_TK” determined by FE–SEM–EDS. Each data point represents the compositional value obtained from a single analysis. (a) Na_2_O vs. TiO_2_ content in Cpx grains. (b) Al_2_O_3_ content vs. Mg#, where Mg# is the atomic ratio of Mg to (Mg + Fe). Notably, the host Cpx grains containing the inclusions (see Fig. 1) exhibited lower Ti and Al contents and a higher Mg# than other Cpx grains in the same section.


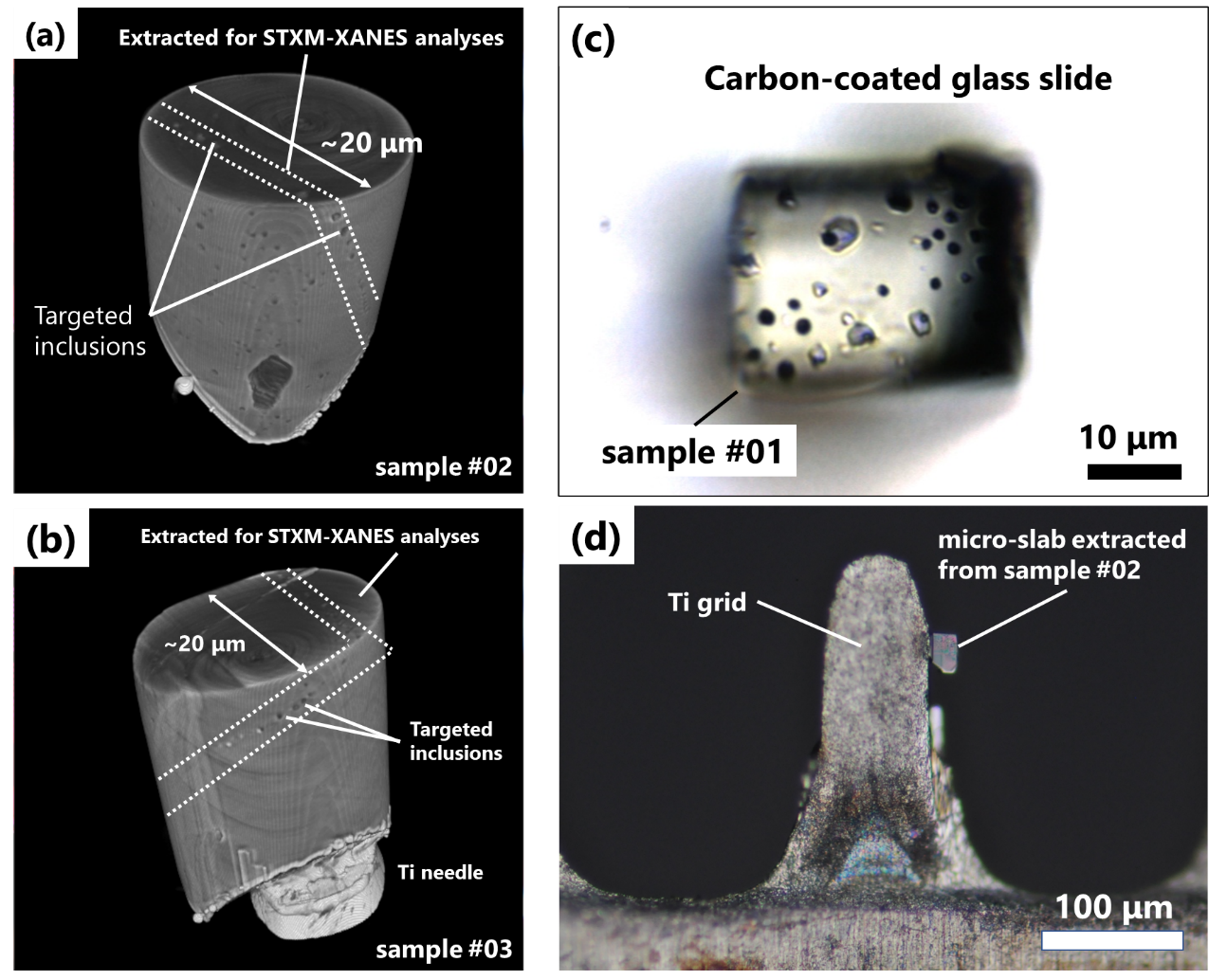


Supplementary Fig. S3. Three-dimensional views of samples (a) #02 and (b) #03. White-dotted lines indicate the regions extracted for STXM–XANES analysis. (c) Photomicrograph of sample #01 mounted on a carbon-coated glass slide. (d) Photomicrograph of the microslab extracted from sample #02.


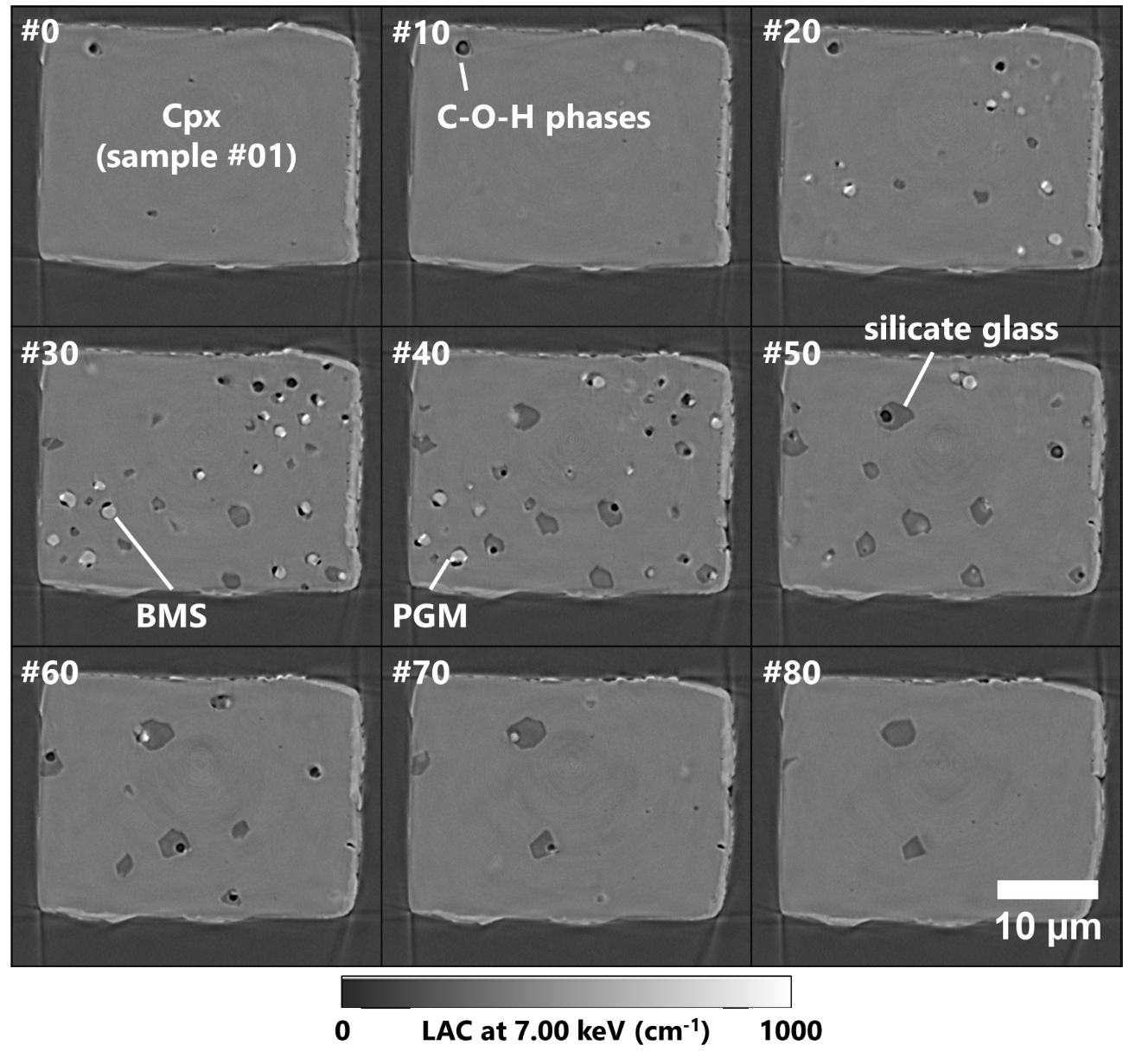


Supplementary Fig. S4. Cross-sectional XnCT images of sample #01. Numbers in each image indicate the relative number of slices. The voxel size of each image was 47.2 nm. Abbreviations: Cpx = clinopyroxene; BMS = base metal sulphides; PGM = platinum-group minerals.


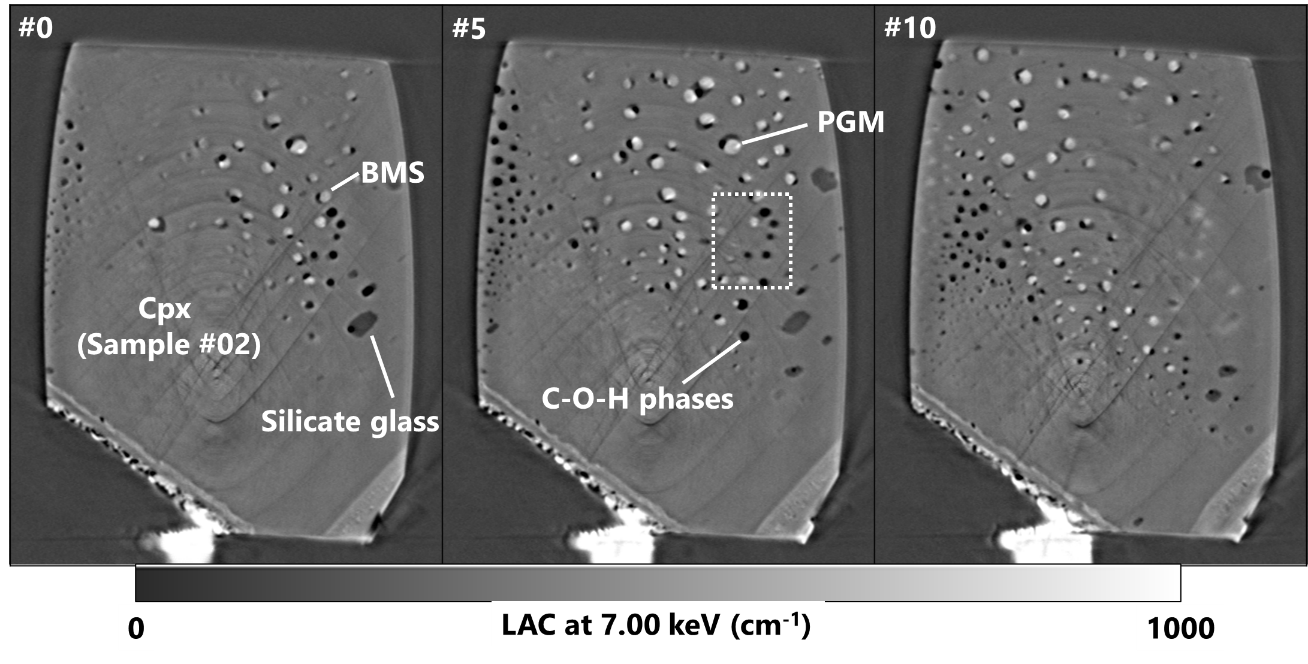


Supplementary Fig. S5. Cross-sectional XnCT images of sample #02. Numbers in each image indicate the relative number of slices. The voxel size of each image was 44.6 nm. Abbreviations: Cpx = clinopyroxene; BMS = base metal sulphides; PGM = platinum-group minerals.


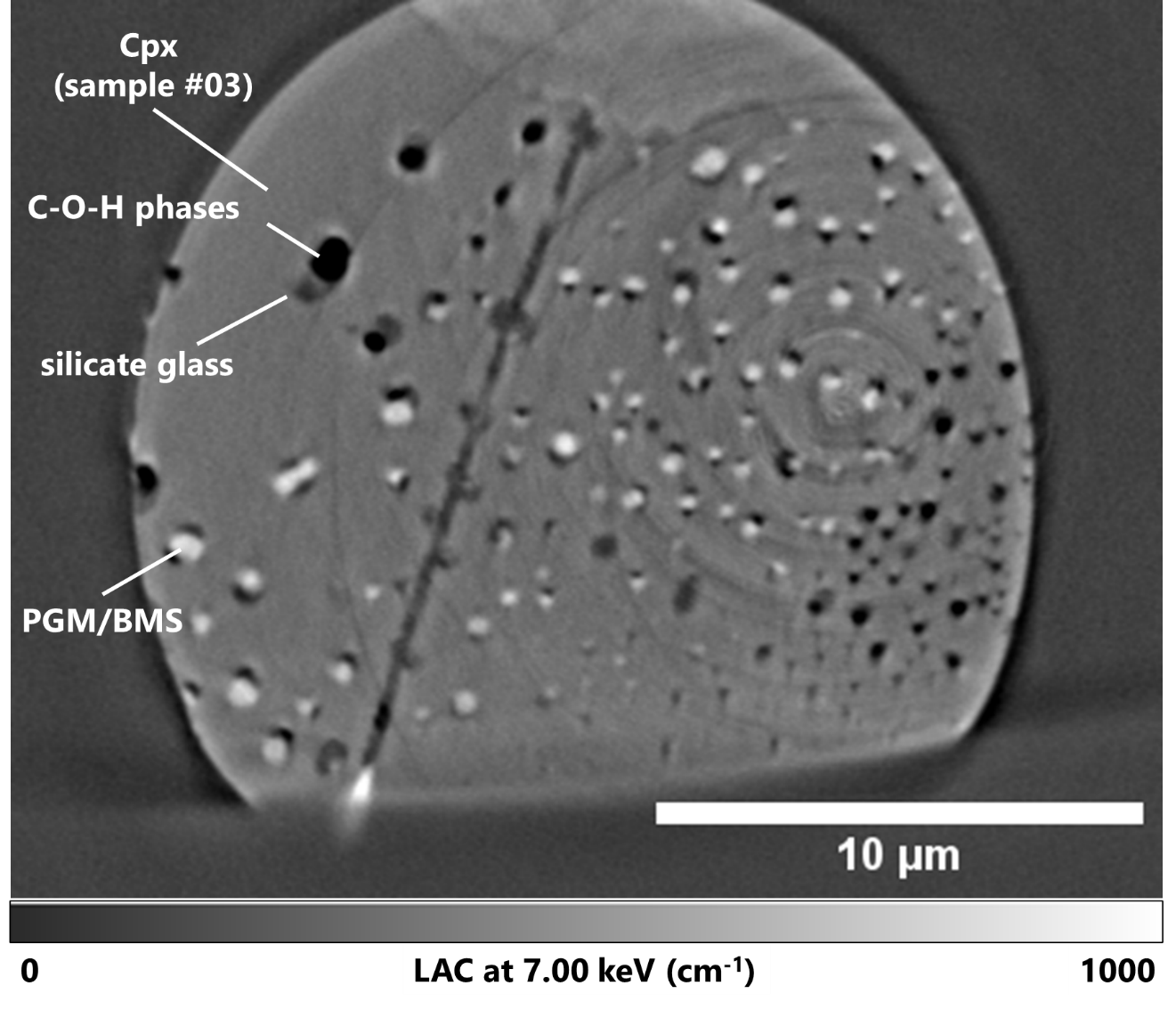


Supplementary Fig. S6. Cross-sectional XnCT images of sample #03. The voxel size of each image was 44.6 nm. Abbreviations: Cpx = clinopyroxene; BMS = base metal sulphides; PGM = platinum-group minerals.


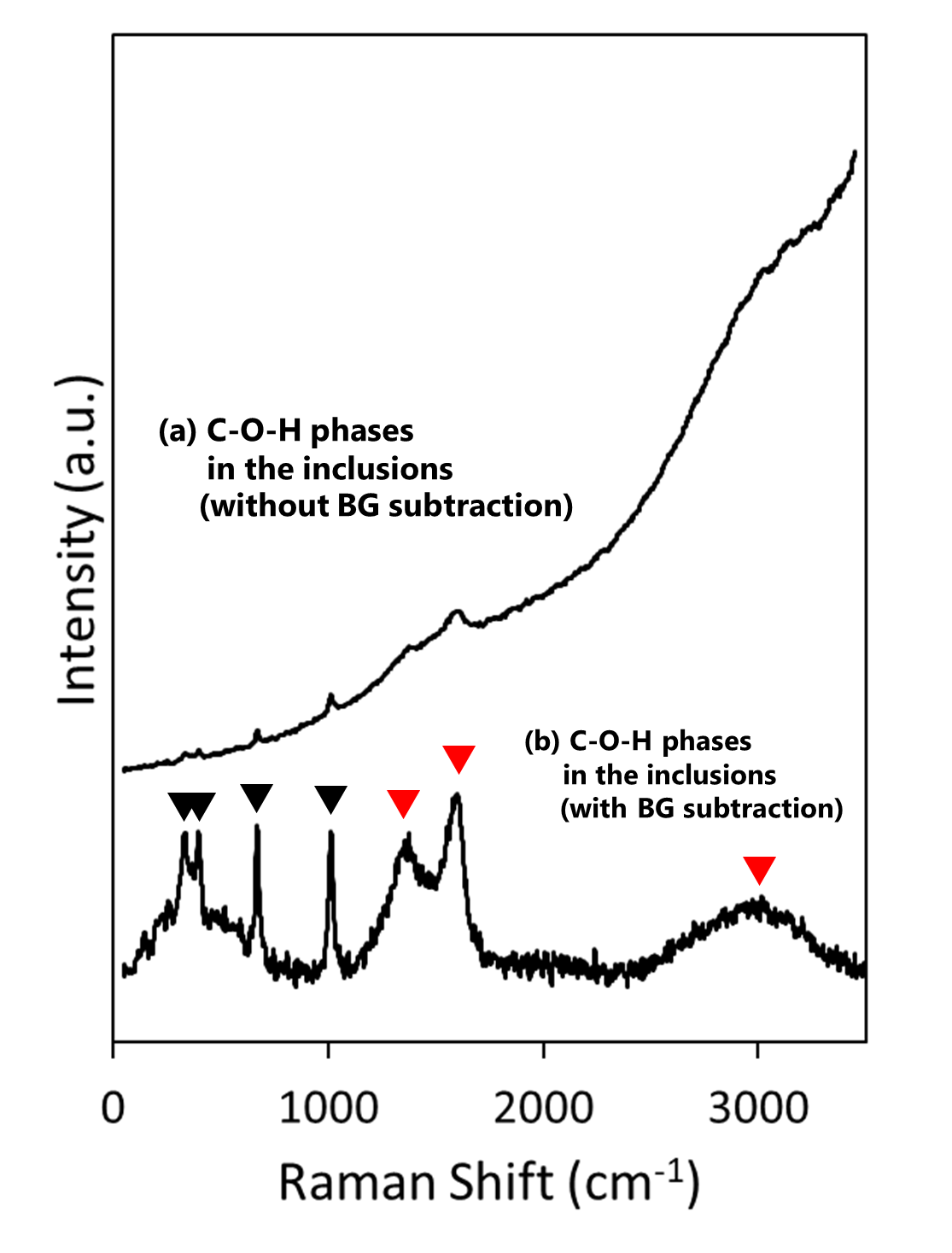


Supplementary Fig. S7. Raman spectra of inclusions in sample #01. (a) Without background (BG) subtraction. (b) With BG subtraction. Red and black triangles indicate peaks derived from the C–O–H phases and host Cpx, respectively. Abbreviations: a.u. = arbitrary units.


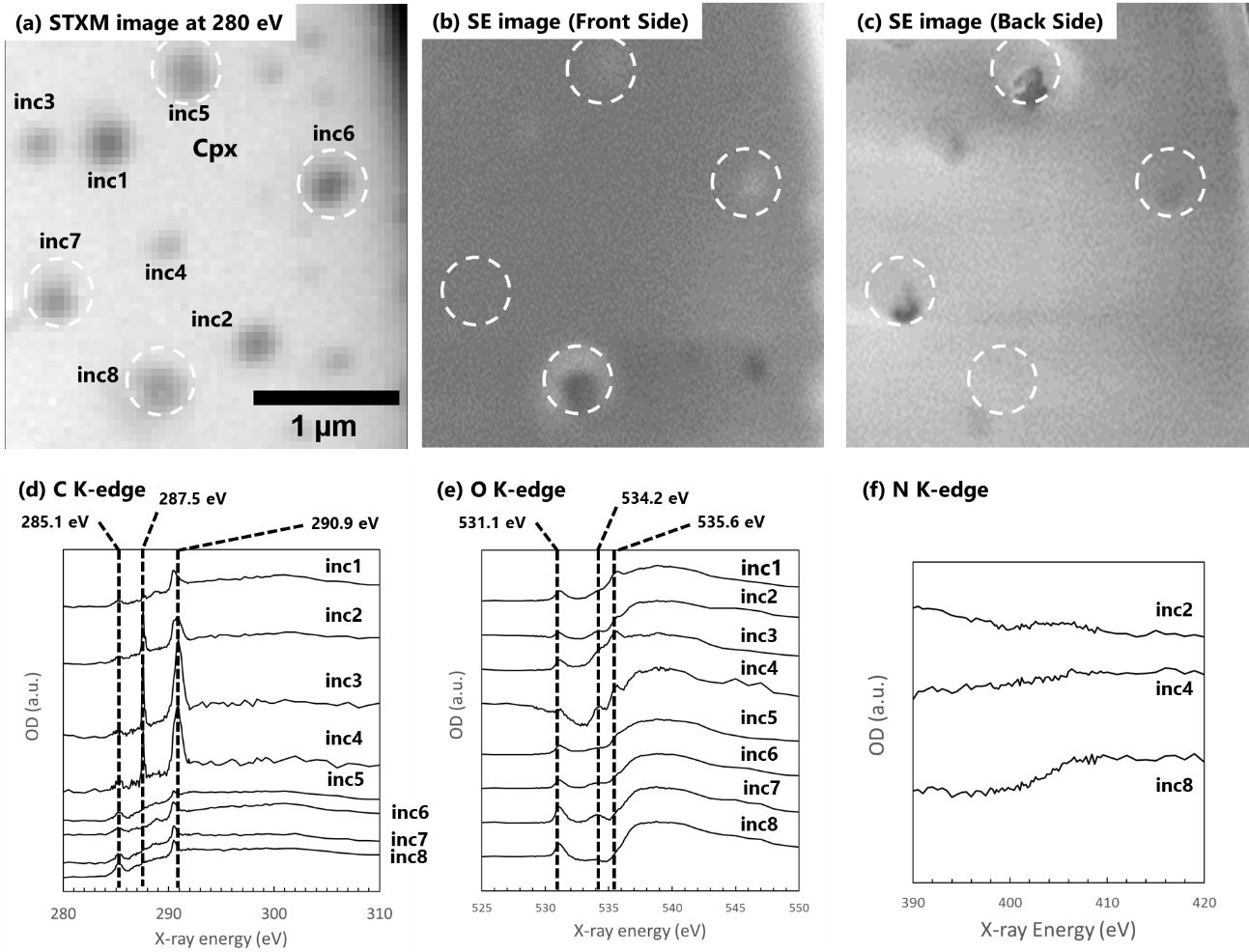


Supplementary Fig. S8. STXM–XANES analysis results of sample #03. (a) STXM image at 280 eV, where contrast corresponds to the degree of absorption. The numbers beside each inclusion correspond to those in (d), (e), and (f). White-dotted circles indicate inclusions that do not exhibit peaks at 287.5 and 290.9 eV and correspond to those in (b) and (c). (b) Frontside and (c) Backside secondary electron (SE) images of the sample surface. Notably, the inclusions indicated by white-dotted circles are exposed on the sample surface. (d) C K-edge XANES spectra. Some inclusions exhibited peaks at 290.5 eV. This peak can be attributed to organic carbonate^23^. This is most likely due to the presence of contaminants on the sample surface, as reported in previous studies^52^. (e) O K-edge XANES spectra. Peaks at 531.1 eV are attributed to oxygen in the silicate glass. Peaks at 534.1 eV are attributed to alcohols/ethers; most likely derived from contaminants, as they are also detected in the Cpx regions. (f) N K-edge XANES spectra. The spectra shown in (d–f) were obtained by subtracting the normalised host Cpx spectrum from that of the inclusions. Abbreviations: OD, optical density; a.u., arbitrary units; Cpx, clinopyroxene; BMS/PGM, base metal sulphides and platinum-group minerals.

**Supplementary Tables**

Supplementary Table S1. Average elemental compositions of Cpx (Fig. 1b), Na-rich Cpx, and Na-poor Cpx.

|  | **Cpx in Fig. 1b** | | **Na-rich Cpx** | | **Na-poor Cpx** | |
| --- | --- | --- | --- | --- | --- | --- |
| (wt. %) | (N = 7) | | (N = 96) | | (N = 83) | |
|  | Ave. | Std. Dev. | Ave. | Std. Dev. | Ave. | Std. Dev. |
| SiO_2_ | 52.15 | 0.64 | 51.94 | 0.91 | 51.90 | 0.78 |
| TiO_2_ | 0.11 | 0.02 | 0.40 | 0.26 | 0.55 | 0.07 |
| Al_2_O_3_ | 4.57 | 0.06 | 4.98 | 0.74 | 3.06 | 0.22 |
| Cr_2_O_3_ | 1.68 | 0.03 | 1.58 | 0.26 | 1.57 | 0.06 |
| FeO | 3.10 | 0.05 | 3.26 | 0.30 | 3.13 | 0.11 |
| MnO | 0.09 | 0.04 | 0.08 | 0.05 | 0.08 | 0.05 |
| MgO | 16.06 | 0.26 | 16.02 | 0.65 | 18.00 | 0.35 |
| CaO | 19.53 | 0.21 | 19.53 | 0.71 | 20.33 | 0.31 |
| Na_2_O | 1.50 | 0.04 | 1.55 | 0.09 | 0.45 | 0.11 |
| Mg# | 90.21 | 0.20 | 89.74 | 0.71 | 91.11 | 0.24 |

Supplementary Table S2. Excitation and detection wavelengths of filters used in wide-field fluorescence microscopy.

| Filter name | Excitation Wavelength [nm] | Detected wavelength [nm] |
| --- | --- | --- |
| D | 355–425 | 470– |
| CFP | 426–446 | 460–500 |
| I3 | 450–490 | 515– |
| N21 | 515.5–560.5 | 590– |
| BGR | B: 390–410  G: 487.5–502.5  R: 560–580 | B: 455–475  G: 515–545  R: 620–660 |
